# Supplementary material for: Induction of Inflammation Disrupts the Negative Interplay between STING and S1P Axis That Is Observed during Physiological Conditions in the Lung
Source: Int J Mol Sci. 2023 May 5;24(9):8303. doi: 10.3390/ijms24098303 (PMC10179278; doi:10.3390/ijms24098303)
Supplement: Supplementary file 1 [file ijms-24-08303-s001.zip › ijms-2369817-supplementary.pdf]

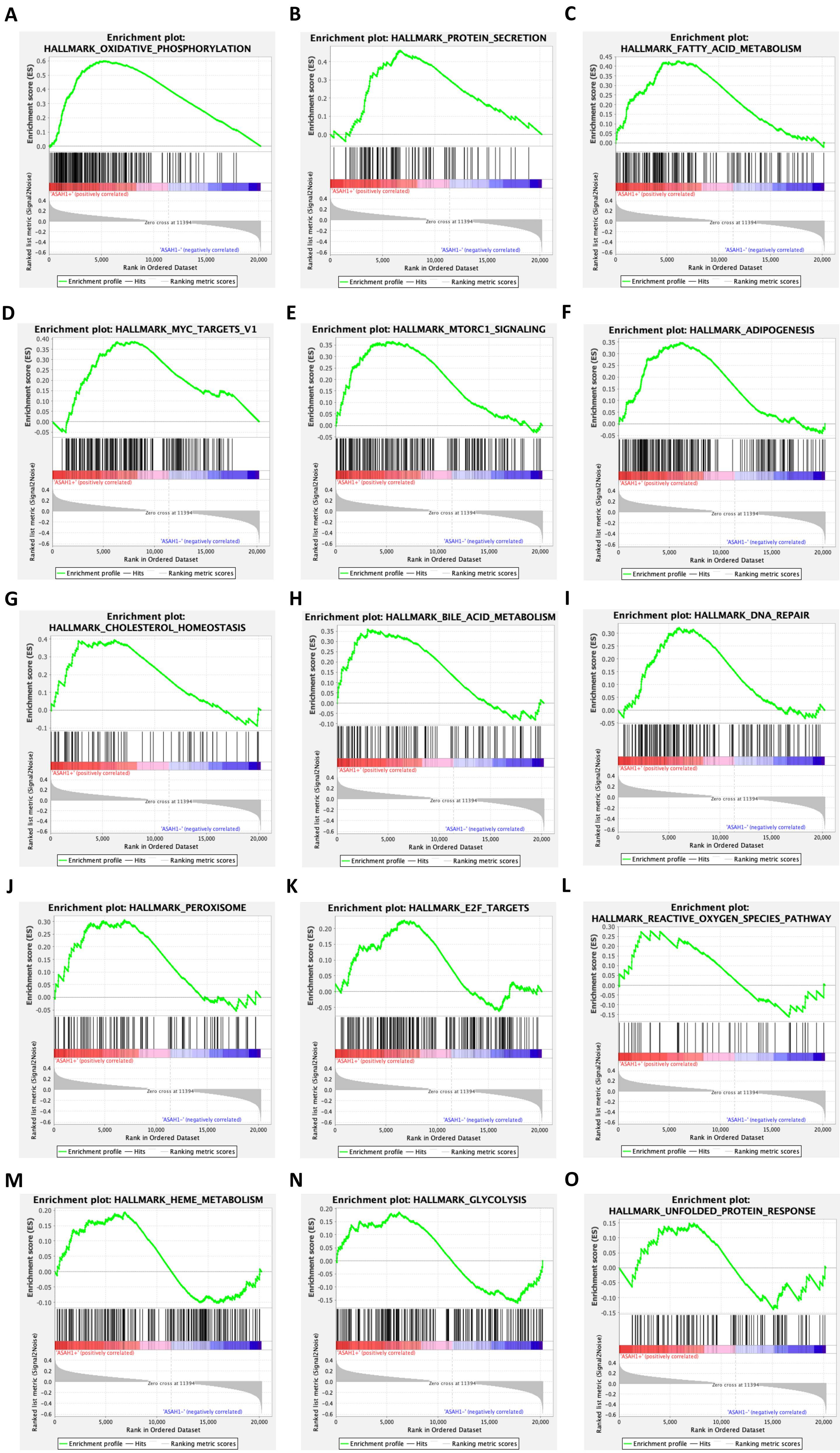


**Supplementary Figure S1**

**Supplementary Figure S1. Gene sets enriched in the phenotype ASAH1+ vs ASAH1- healthy lung tissues.** GSEA analysis was performed according to the *hallmark* database; the enriched gene sets are: oxidative phosphorylation (**A**), protein secretion (**B**), fatty acid metabolism (**C**), myc target v1 (**D**), mtorc1 signaling (**E**), adipogenesis (**F**), cholesterol homeostasis (**G**), bile acid metabolism (**H**), DNA repair (**I**), peroxisome (**J**), E2F targets (**K**), reactive oxygen species pathway (**L**), heme metabolism (**M**), glycolysis (**N**), unfolded protein response (**O**).

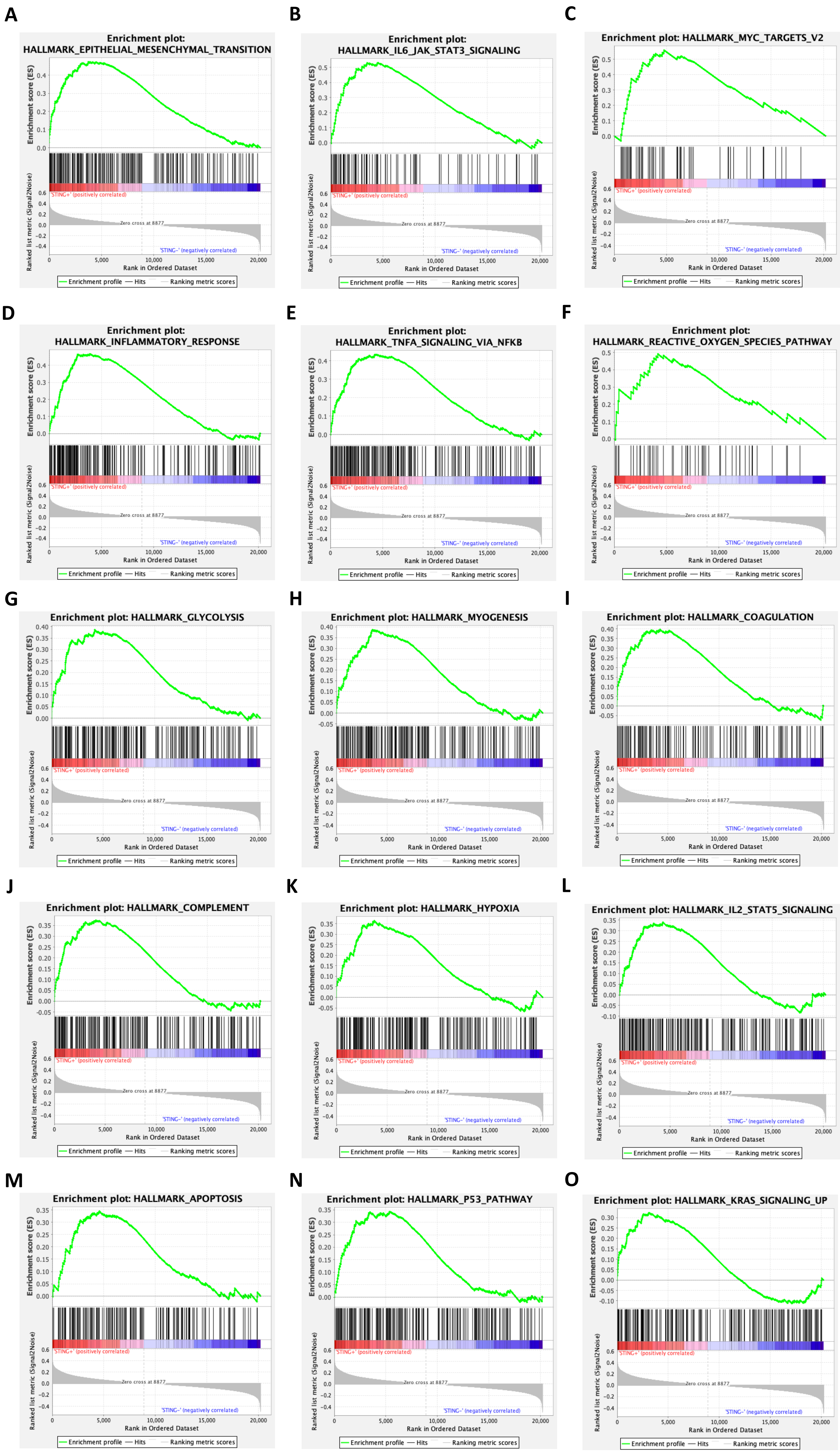


**Supplementary Figure S2**

**Supplementary Figure S2. Gene sets enriched in the phenotype STING+ vs STING- healthy lung tissues.** GSEA analysis was performed according to the *hallmark* database; the enriched gene sets are: epithelial mesenchymal transition (**A**), IL6 JAK STAT3 signaling (**B**), myc targets v2 (**C**), inflammatory response (**D**), TNFA signaling via NF-κB (**E**), reactive oxygen species pathway (**F**), glycolysis (**G**), myogenesis (**H**), coagulation (**I**), complement (**J**), hypoxia (**K**), IL2 STAT5 signaling (**L**), apoptosis (**M**), P53 pathway (**N**), KRAS signaling UP (**O**).

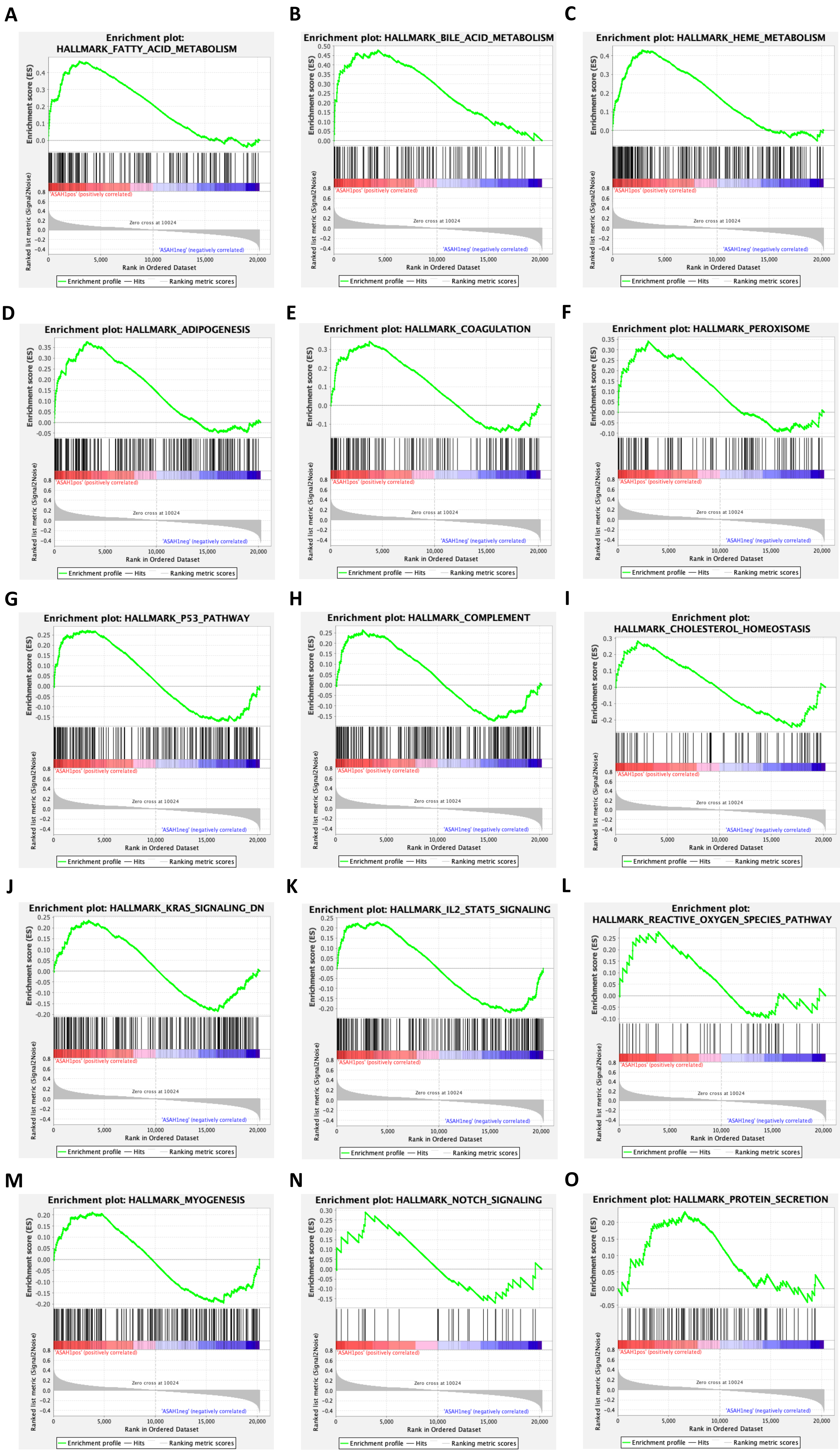


Supplementary Figure S3

**Supplementary Figure S3. Gene sets enriched in the phenotype ASAH1+ vs ASAH1- tumor lung tissues.** GSEA analysis was performed according to the *hallmark* database; the enriched gene sets are: fatty acid metabolism (**A**), bile acid metabolism (**B**), heme metabolism (**C**), adipogenesis (**D**), coagulation (**E**), peroxisome (**F**), P53 pathway (**G**), complement (**H**), cholesterol homeostasis (**I**), KRAS signaling DN (**J**), IL2 STAT5 signaling (**K**), reactive oxygen species pathway (**L**), myogenesis (**M**), NOTCH signaling (**N**), protein secretion (**O**).

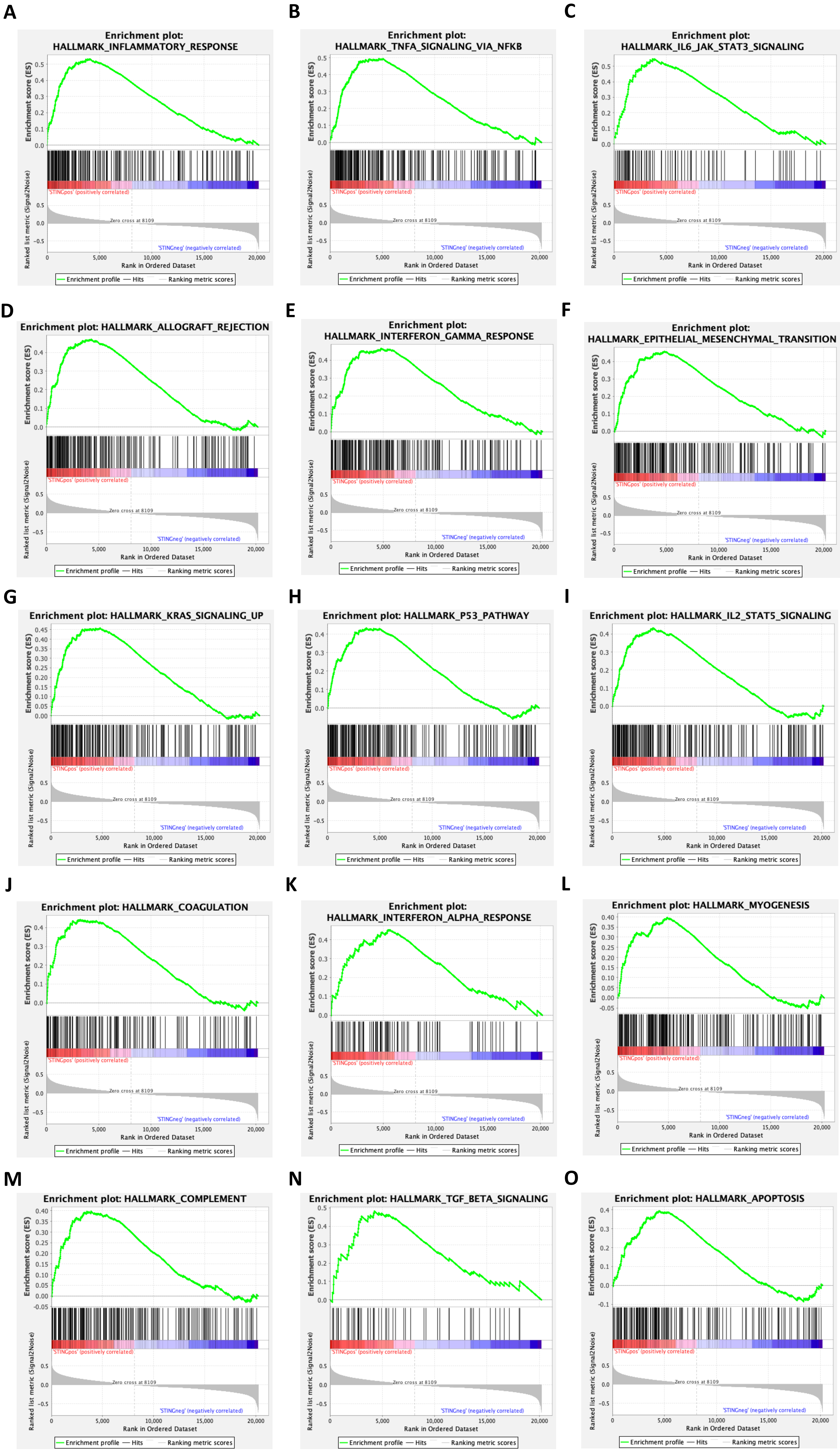


Supplementary Figure S4

**Supplementary Figure S4. Gene sets enriched in the phenotype STING+ vs STING- tumor lung tissues.** GSEA analysis was performed according to the *hallmark* database; the enriched gene sets are: inflammatory response (**A**), TNFA signaling via NF-κB (**B**), IL6 JAK STAT3 signaling (**C**), allograft rejection (**D**), interferon gamma response (**E**), epithelial mesenchymal transition (**F**), KRAS signaling UP (**G**), P53 pathway (**H**), IL2 STAT5 signaling (**I**), coagulation (**J**), interferon alpha response (**K**), myogenesis (**L**), complement (**M**), TGF beta signaling (**N**), apoptosis (**O**).
